# Ion Torrent PGM as Tool for Fungal Community Analysis: A Case Study of Endophytes in *Eucalyptus grandis* Reveals High Taxonomic Diversity

**DOI:** 10.1371/journal.pone.0081718

**Published:** 2013-12-16

**Authors:** Martin Kemler, Jeff Garnas, Michael J. Wingfield, Marieka Gryzenhout, Kerry-Anne Pillay, Bernard Slippers

**Affiliations:** 1 Department of Genetics, Forestry and Agricultural Biotechnology Institute (FABI), University of Pretoria, Pretoria, South Africa; 2 Department of Zoology and Entomology, Forestry and Agricultural Biotechnology Institute (FABI), University of Pretoria, Pretoria, South Africa; 3 Department of Plant Sciences, University of the Free State, Bloemfontein, South Africa; Argonne National Laboratory, United States of America

## Abstract

The Kingdom Fungi adds substantially to the diversity of life, but due to their cryptic morphology and lifestyle, tremendous diversity, paucity of formally described specimens, and the difficulty in isolating environmental strains into culture, fungal communities are difficult to characterize. This is especially true for endophytic communities of fungi living in healthy plant tissue. The developments in next generation sequencing technologies are, however, starting to reveal the true extent of fungal diversity. One of the promising new technologies, namely semiconductor sequencing, has thus far not been used in fungal diversity assessments. In this study we sequenced the internal transcribed spacer 1 (ITS1) nuclear encoded ribosomal RNA of the endophytic community of the economically important tree, *Eucalyptus grandis*, from South Africa using the Ion Torrent Personal Genome Machine (PGM). We determined the impact of various analysis parameters on the interpretation of the results, namely different sequence quality parameter settings, different sequence similarity cutoffs for clustering and filtering of databases for removal of sequences with incomplete taxonomy. Sequence similarity cutoff values only had a marginal effect on the identified family numbers, whereas different sequence quality filters had a large effect (89 vs. 48 families between least and most stringent filters). Database filtering had a small, but statistically significant, effect on the assignment of sequences to reference sequences. The community was dominated by Ascomycota, and particularly by families in the Dothidiomycetes that harbor well-known plant pathogens. The study demonstrates that semiconductor sequencing is an ideal strategy for environmental sequencing of fungal communities. It also highlights some potential pitfalls in subsequent data analyses when using a technology with relatively short read lengths.

## Introduction

Fungi are an enormously diverse group of organisms, exhibiting great environmental tolerance and plasticity and are abundant across a wide range of habitats [1], [2]. They play an essential role as decomposers, plant and insect associates, pathogens and providers of ecosystem services. For these reasons fungi are increasingly being considered in studies of terrestrial biodiversity (e.g., soil diversity [3], indoor diversity [4], plant associated diversity [5]). This trend is greatly facilitated by recent advances in sequencing technology that have made it feasible to capture the often hidden microbial diversity [6], [7]. Environmental sequencing has revealed that in many cases, the vast majority of microbes are uncultivable using traditional methods [8]. Consequently, they have effectively been invisible to past generations of microbiologists.

Despite rapid advances in next generation sequencing technologies (NGS) and exponential reductions in per base pair costs, important limitations persist. Paramount among the current challenges is data processing, interpretation, and archiving. Bioinformatics is confronted with enormous volumes of sequence information, and must deal with issues of variable read length and quality, among other technical issues [9]–[13]. More importantly, databases such as GenBank have become the *de facto* repositories for environmental sequencing data. Unfortunately a considerable proportion of uploaded reads carry minimal taxonomic information [14], limiting their utility to comparative biology. Taxa that have only a small percentage of archived specimen-based sequences linked to a taxonomic name compared to the representatives from environmental sequencing without proper taxonomic affiliation, therefore might query against these unknowns even though there is potential taxonomic information available.

Endophytic fungi, defined here as organisms that live within plant tissue without visible symptoms of disease or decay, appear to contribute to a significant proportion of the already astonishing fungal diversity, but their characterization is problematic [15], [16]. Culture-derived approaches, the dominant method used in endophyte studies, suffer from organismal bias and are far too labor-intensive to adequately assess highly diverse fungal communities. Direct DNA clone sequencing avoids culturing bias and has the potential to detect rare taxa but is expensive and time consuming. Thus, while these tools have initially exposed the diverse nature of fungi from environmental samples, they face major limitations in cost and labor for the assessment of these plant-associated fungal communities.

Direct assessments of environmental fungal DNA using next generation sequencing have changed our understanding of microbial diversity. Endophyte diversity, the focus of this paper, may have been underestimated by orders of magnitude [5], [17], [18]. Although NGS approaches have increased the information obtained on microbial communities from various biota by decreasing the cost per base pair, these approaches still remain constrained by high overall costs linked to library preparation and sequencing. This limits their availability to institutions and countries with a fair amount of funding.

One of the platforms that have entered the competition of NGS technologies is semiconductor sequencing as implemented in the Ion Torrent Personal Genome Machine (PGM). By placing a micro-well layer above a microchip, Ion Torrent sequencing combines sequencing-by-synthesis with semiconductor technology. Single-stranded DNA and polymerase are combined in each micro-well and flooded with the four individual nucleotides. Incorporation of a nucleotide into the growing DNA strand releases a proton resulting in a change in pH. A sensor layer beneath each well converts this pH change into voltage, which is subsequently translated into sequence data [19]. When first introduced, the relatively short read-lengths of this technology precluded its routine use in metagenomic studies, as was also true for other NGS technologies. However, data output and read length has increased rapidly to 200 bp as of early 2012 and at the time of writing the sequencing chemistry is able to sequence up to 400 bp (http://www.iontorrent.com/). Sequence output is likewise increasing with the development of new chips. The volume of sequence output (400 Mb; 454 FLX Titanium: 400 Mb), increasing read lengths (400 bp; 454 FLX Titanium: 400 bp), short runtime (4 hrs.; 454 FLX Titanium: 10 hrs.), similar error rates when compared with 454 pyrosequencing (1%) and the relatively low price per Mb ($1.20; 454 FLX Titanium: $12) [20], [21], make semiconductor sequencing a technology practical for metagenomic studies. However, this technology has thus far only been applied successfully to study the composition of prokaryotic communities [22], [23].

The goal of this paper is to examine patterns of endophytic fungal diversity of commercially planted *Eucalyptus grandis* applying semiconductor sequencing on the Ion Torrent PGM. Firstly, we describe richness, diversity and composition in these tree-associated fungi. Secondly, we systematically examine effects of data processing choices, related to quality control, operational taxonomic unit (OTU) grouping and taxonomic assignment on the characterization of these complex communities.

## Materials and Methods

### Ethics statement

The study did not include endangered or protected species. Samples were obtained in agreement with members of the Tree Protective Co-operative Program (TPCP; www.fabinet.up.ac.za/research/tpcp) and no special permission was required.

### DNA amplification and sequencing

DNA used in the amplification of ITS1 region of the rDNA was sampled from three individual *Eucalyptus grandis* trees from a plantation in Mtubatuba (KwaZulu-Natal, South Africa, S 28.498 E 32.165, 33 meters above sea level). DNA was isolated directly from plant material, which included 52 leaf, 42 petiole, 39 twig, and 14 trunk increment core samples (each 5×5 mm) using the Zymo plant/seed extraction Kit™ (Zymo Research, Irvine, USA). The samples were surface-sterilized by placing them for 3 min in 10% hydrogen peroxide with subsequent washing for 1 min in ddH_2_O. Surface sterilization is not strictly necessary for studies of direct sequencing of fungal endophytes (surface washing should suffice) but the samples were also used in a study comparing culture-dependent techniques, reported elsewhere (Gryzenhout, *in prep.*). PCR amplifications were performed using the primers ITS1F [24] and ITS2 [25] with the sequencing adapters trp1 and A attached (Life Technologies, Carlsbad, USA). All amplifications were performed using a standard PCR protocol (3 min at 96°C; 30 cycles with 30 sec at 96°C, 45 sec at 45°C, and 1 min at 72°C each cycle; 7 min at 72°C). In order to remove excess primer, electrophoresis was performed on a 1% agarose gel, bands were cut out in a region between ∼100–400 bp and subsequently purified using the Qiagen Gel Purification Kit (Qiagen, Hilden, Germany). Purified samples were pooled and sequenced on an Ion Torrent PGM (Life Technologies, Carlsbad, USA) using the Ion PGM 200 Sequencing Kit and the Ion 316 Chip Kit. The resulting sequence information was submitted to the Short Read Archive at the European Nucleotide Archive (ERP002241).

### Sequence analysis

The Ion Torrent fastq file was converted into a fasta and a quality file using the script fastq.info in Mothur v.1.25.1 [26]. Unless otherwise stated the QIIME 1.5.0 framework was used for subsequent sequence data analyses [27].

Few published studies have used the Ion Torrent PGM to characterize diversity and composition in environmental samples [22], [23] and there are numerous data processing choices that must be made with the potential to influence the results. Minimal filtering was performed for all sequences by removing all that were shorter than 200 bp or had an average quality score below a Phred quality score of 20.

We varied two main aspects of sequence post-processing for comparison purposes: 1) the presence and sequence quality of the sequenced reverse primer used to amplify the ITS region in our environmental samples; and 2) the stringency of the clustering algorithm used to group similar sequences into operational taxonomic units. We then compared sequence analysis across these treatments and measured the effects on the number of OTUs and their taxonomic assignment. We used three parameter settings with different levels of stringency related to the presence versus absence and sequence integrity of the reverse primer. In the most strict parameter setting (‘perfect match’) only sequences with a perfect matching reverse primer were retained. In the second setting (‘fuzzy match’) the reverse primers were allowed to have a mismatch of 3 bp to accommodate differences in the template DNA as well as minor sequencing errors. For the least stringent parameter setting (‘no primer’) all sequences were retained whether or not the reverse primer sequence was present. In all quality treatments sequences were truncated at the end of the forward primer and at the beginning of the reverse primer sequence, where present (FASTA files for all three quality filtering settings available from the Dryad Digital Repository: http://dx.doi.org/10.5061/dryad.51rf8). For the sequences resulting from the three parameter settings we calculated the mean sequence quality score per nucleotide position using QIIME as well as the GC content per base position using FastQC (http://www.bioinformatics.bbsrc.ac.uk/projects/fastqc).

Assigning sequences to groups that approximate species is critical in understanding the biological diversity revealed by environmental sequencing. As such, we chose to compare biodiversity estimates and OTU identities at 90%, 95%, and 97% sequence similarity levels using uclust v1.2.22q [28] as implemented in QIIME with sequences sorted according to their abundance. The most abundant sequence for each OTU was chosen as a representative sequence. OTU accumulation curves by sequence count were created for each of the different quality filtering and OTU clustering approaches, using a random bootstrapping approach (100 replicates per sampling point) without replacement and excluding singleton OTUs.

Taxonomic assignment of the representative sequence was performed using BLAST [29] against a locally downloaded UNITE database (released: 13.04.2012; including fungal ITS data from the International Nucleotide Sequence Databases) [30], which only includes ITS data for fungi. Due to the shorter expected read lengths of the Ion Torrent PGM as well as the potential inaccuracy in assigning OTUs to species [31] we restricted taxonomic assignment to the family level or higher. Clearly, taxonomic assignment at the genus or species-level would be preferable, but fungal DNA databases are currently inadequate and our read-lengths too short to ensure definitive hits at lower taxonomic levels. A specific problem with current databases is the accumulation of sequences, from mainly NGS approaches, with taxonomic description such as “uncultured Fungus”, “uncultured Basidiomycete”, or “uncultured ectomycorrhiza” [14]. To understand the effect of this on the taxonomic assignment on our dataset we compared OTUs against the unfiltered database as well as against a filtered version that did not include sequences with insufficient taxonomic description. Hits against both the filtered and unfiltered database were considered only when sequence similarity exceeded 90% and had an e-value ≥1e^−30^; such hits were not filtered for query coverage. In order to reveal how database filtering influenced support for given taxonomic assignments, e-values were compared with a full three-way ANOVA using the predictor variables for database filtering (y/n), reverse primer filtering (‘perfect match’, ‘fuzzy match’ and ‘no primer’), and cutoff percentage for OTU grouping (90, 95 and 97%). The response variable was log-transformed to meet model assumptions. We performed univariate Chi-squared tests to examine the importance of filtering method and sequence similarity grouping criteria on estimated OTU and family richness. All data exploration, modeling and statistics were performed in R v3.0.0 [32]

The ‘no primer’ parameter settings resulted in nearly twice as many families as the ‘perfect match’ quality filtering. In order to elucidate the difference between the OTUs, the respective representative OTU sequences from the ‘no primer’ settings for the 95% sequence similarity were compared against GenBank (megablast). The five best hits were downloaded and the sequences were aligned using MAFFT v7.045b [33], [34].

## Results

### DNA sequencing

The sequencing run resulted in 2,394,051 sequences and 328.01 Mbp, of which 208.50 Mbp had a Phred quality score higher than 20. Prior to quality filtering, the average read length was 137 bp and the longest sequence had 365 bp. A frequency histogram across a range of sequence lengths showed a bimodal distribution with peaks at about 50–90 bp and 270 bp (Figure S1)

### Sequence analysis

The number of sequences differed vastly between the different quality filtering parameter settings (Table 1, Figure S2). The most liberal filtering (‘no primer’) method resulted in 461,596 sequences (min./mean/max. 157/227/345 bp), the number of sequences dropped to 168,775 (min./mean/max. 156/220/291 bp) in the ‘fuzzy match’ approach and to 86,556 (min./mean/max. 157/217/291 bp) in the ‘perfect match’ approach.

**Table 1:** Overview of the results of the effects of the different quality and filtering approaches.

		90% OTU similarity			95% OTU similarity			97% OTU similarity		
Quality filtering	Number of sequences/percentage of raw sequences	OTUs/w/o singletons	BLAST hits for filtered database	BLAST hits for unfiltered database*	OTUs/w/o singletons	BLAST hits for filtered database	BLAST hits for unfiltered database*	OTUs/w/o singletons	BLAST hits for filtered database	BLAST hits for unfiltered database*
‘no primer’	461596/19.28%	1566/1151	907	975 (387)	8119/4975	4260	4525 (1669)	22190/11321	10221	10645 (3342)
‘fuzzy match’	168775/7.05%	391/284	230	253 (131)	1426/932	826	863 (349)	4332/2415	2191	2254 (760)
‘perfect match’	86556/3,62%	295/207	159	180 (99)	800/486	422	439 (193)	2145/1154	1034	1071 (398)

*numbers in parentheses represent BLAST hits to database sequences with insufficient taxonomy.

**Figure 1:**
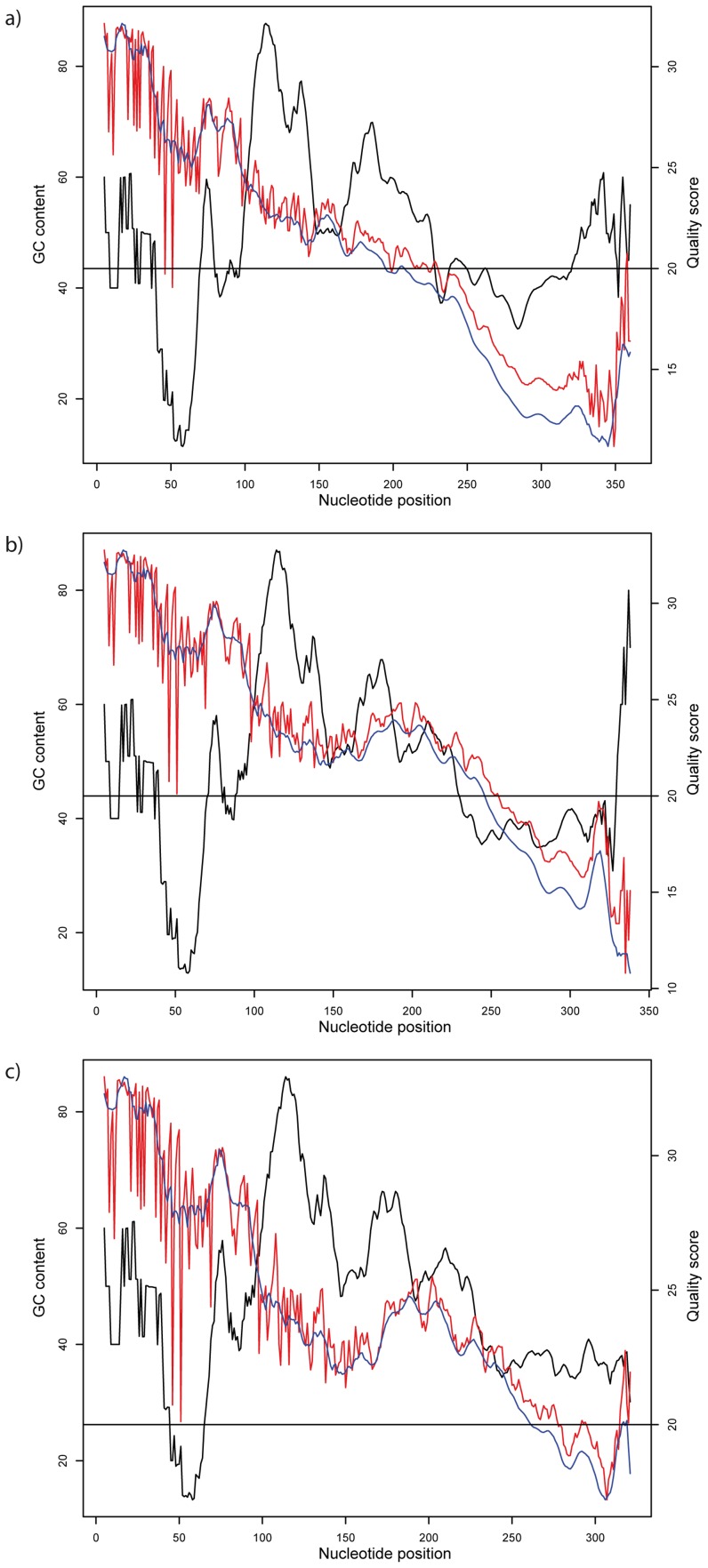
Sequence quality and GC content for the different parameter settings. Black: average GC content using a sliding window approach (window size 10 bp); red: average sequence quality per base; blue: average sequence quality per base using a sliding window approach (window size 10 bp). a) ‘no primer’; b) ‘fuzzy match’; c) ‘perfect match’. Full sequence lengths before quality trimming is shown.

The average sequence quality declined in all three approaches along sequence lengths, dropping under the average quality score Q20 at ∼200 bp in the ‘no primer’ approach and ∼250 bp in the more stringent filtering approaches (Figure 1 & Figure S3). Average sequence quality showed a dip between 50 and 70 bp in all filtering treatments, with average sequence quality dropping under Q20 at 45 and 50 bp. Average GC content varied considerably in different regions of the sequence with minimum values between 40 and 70 bp and a maximum between 100 and 150 bp (Figure 1). Sequence quality rose at about 170 bp up to around 200 bp in a region that also showed a higher GC content, though only for the two more stringent primer filtering treatments. GC content increased in the ‘no primer’ approach, but sequence quality did not rise in these latter positions.

**Figure 2:**
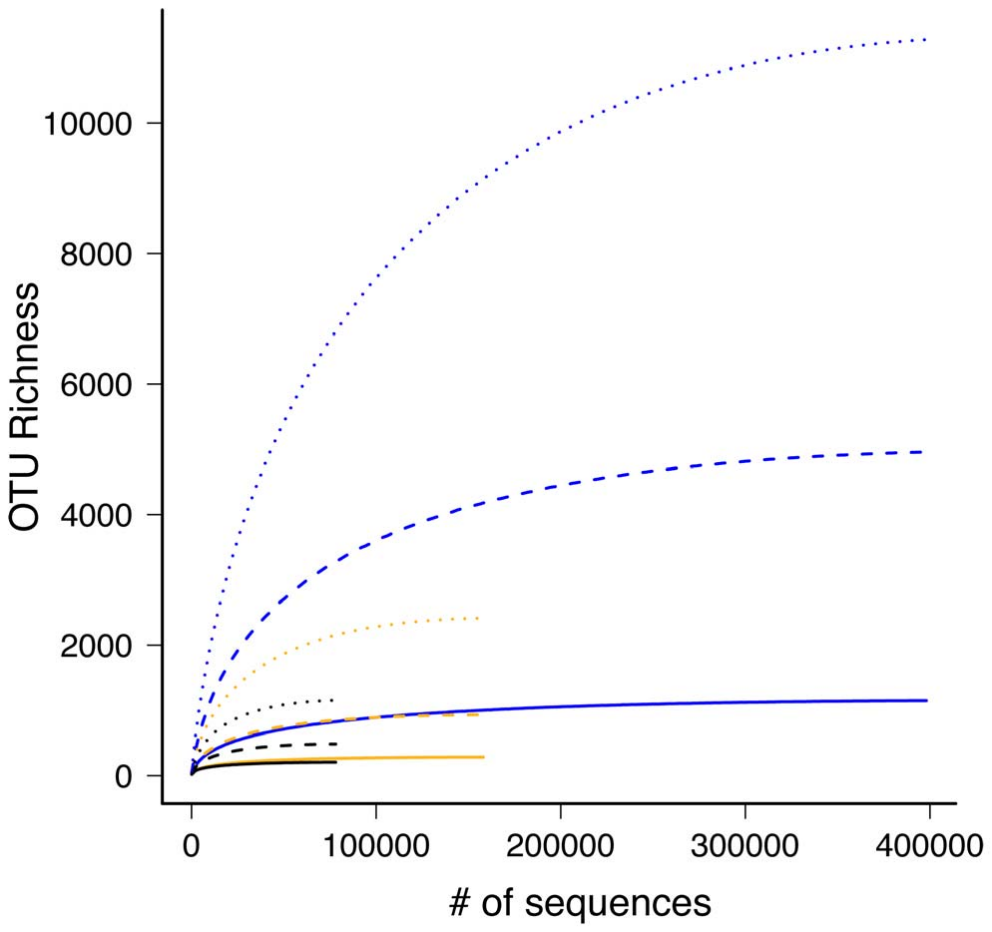
OTU accumulation curve. Accumulation curve for OTUs of endophytic fungi of *Eucalyptus* for the parameter settings (black: ‘perfect match’; orange: ‘fuzzy match’; blue: ‘no primer’) and OTU sequence similarity values (solid: 90%; dashed: 95%; dotted: 97%). See Figure S4 for 95% CIs.

Quality filtering strongly influenced OTU clustering; although more sequences always resulted in more OTUs (Table 1), the relationship between the number of sequences and the number of OTUs clustered by the different sequence similarity cutoffs was non-trivial. Although the number of sequences was fourfold higher in the ‘no primer’ quality filtering compared to the ‘perfect match’ filtering, the amount of OTUs between these two approaches was five (90% sequence similarity) to ten (95% & 97% sequence similarity) times higher. Differences in the number of OTUs between the different sequence similarity values (90%, 95%, and 97%) within a single filtering treatment existed (Table 1). For each treatment, the slope of accumulation of observed OTUs in the accumulation curves decreased with increased sampling (Figure 2 & Figure S4).

### Taxonomic assignment and effect of database curation

Excluding insufficiently identified sequences from the reference database reduced the number of OTUs assigned, but on the other side BLAST hits to database sequences with insufficient taxonomy increased disproportionately (Table 1). Clearly many of our OTUs are more similar to taxonomically unassigned sequences in the public database than to the sequences linked to taxonomic identities. The comparison of e-values showed statistically significant differences between filtered and unfiltered database (ANOVA F = 48.5, df = 1,72620, P<0.0001; Figure S5). However, mean e-values were quite similar between the two methods (filtered versus unfiltered) and given the added value or more reliable family assignment (and thus more useful biological information) further analyses on family richness were restricted to the dataset from the filtered database.

### Fungal community

**Figure 3:**
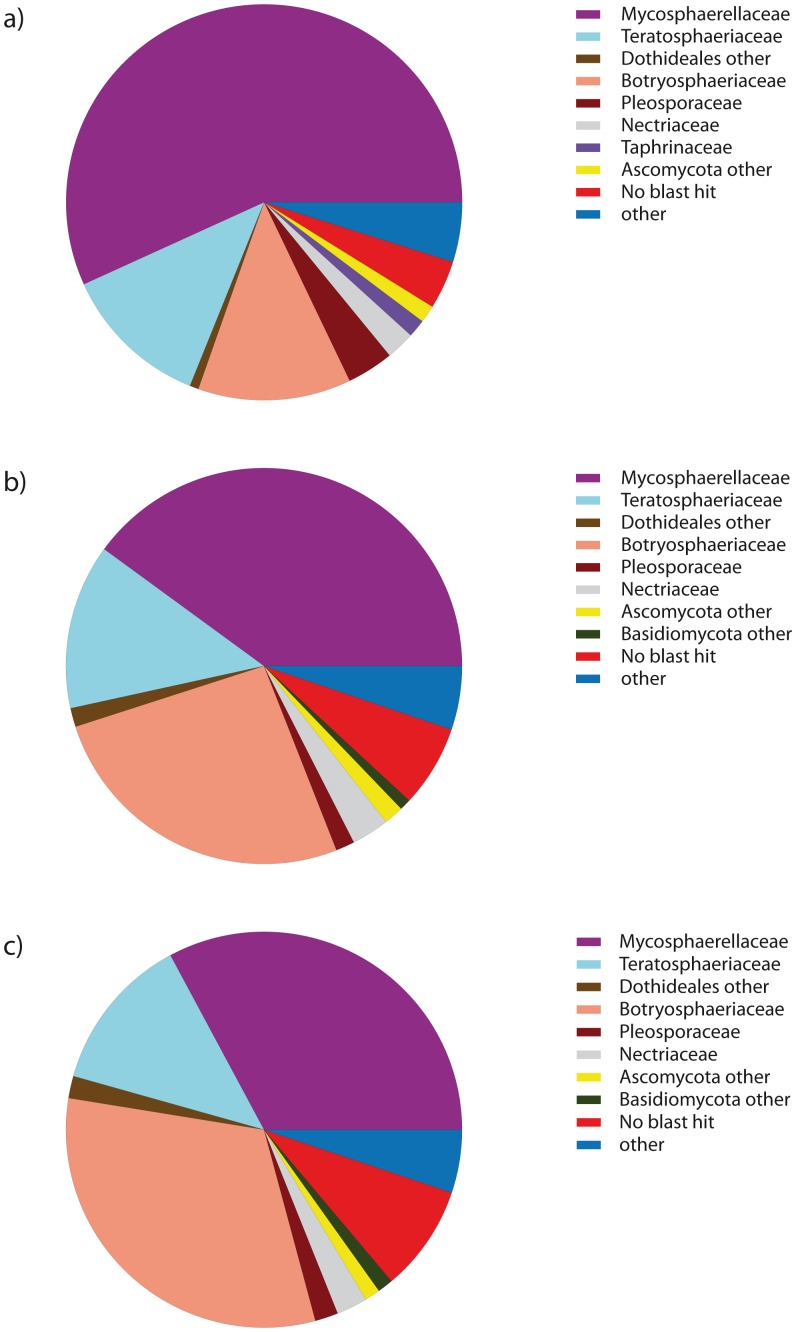
Top fungal taxa recovered. The nine most abundant taxa for the three different parameter settings at a 95% OTU sequence similarity value. a) ‘no primer’; b) ‘fuzzy match’; c) ‘perfect match’.

All three quality filtering and sequence similarity approaches resulted in similar composition of dominant fungal families (Figure 3). The greatest number of OTUs and the largest number of sequences were for the families Botryosphaeriaceae, Mycosphaerellaceae, Nectriaceae, Pleosporaceae, and Teratosphaeriaceae (Table S1). The one exception in the dominant families was the Taphrinaceae, which had high number of sequences in the ‘no primer’ settings but represented a small proportion of sequences in the other two treatments. Additionally, many taxa that include yeasts and dimorphic fungi were recovered (Table S1), some of which had relatively high sequence and OTU richness (e.g., Sporidiobolales and Filobasidiaceae).

**Figure 4:**
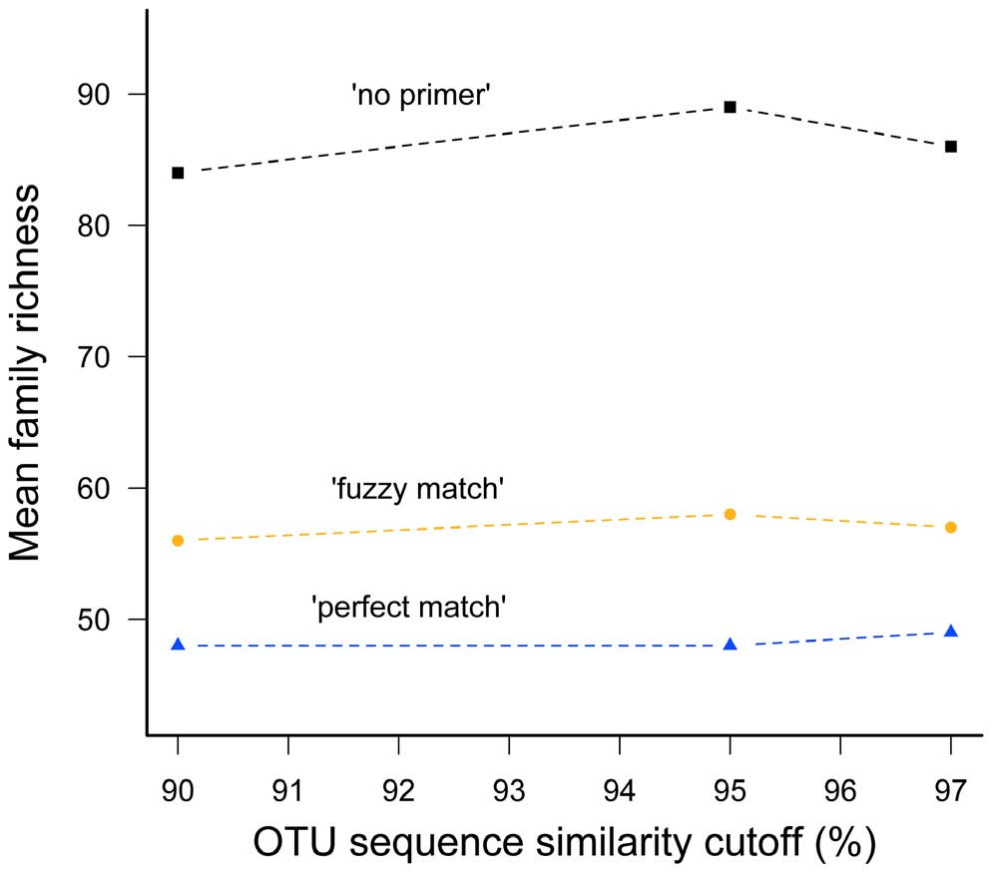
Mean family richness. Mean family richness for the parameter settings (‘perfect match’, ‘fuzzy match’, ‘no primer’) and OTU sequence similarity values (90%, 95%, 97%).

Family richness differed by quality filtering parameter settings (χ^2^ = 12.4, df = 2, p<0.002), but not by sequence similarity values (χ^2^ = 0.4, df = 2, p = 0.98; Table S2). Family composition in the most stringent quality parameter settings always comprised a subset of that recovered under the less stringent settings (Table S1). Differences in community composition between treatments resulted mainly from additional families with few sequences and/or OTUs. The ‘no primer’ approach always resulted in substantially more fungal families compared to the ‘perfect match’ approach (Figure 4 & Table S2). Although many of these families were only recovered with low read abundance the majority showed high similarity to sequences from GenBank in the alignments. In 14 cases, we identified a matching reverse primer sequence in the downloaded sequences, but the Ion Torrent reads were too short to obtain sequence information in this DNA region. In two other cases we identified a mismatch in the primer sequence and in the remaining 12 alignments we could not find the reverse primer sequence at all (Table S3).

## Discussion

The sequencing of the ITS1 region of the rDNA of endophytic fungi from *Eucalyptus grandis* using the Ion Torrent PGM resulted in over 2.3 million sequences. Even though quality filtering discarded the majority of reads, a large number of high quality reads remained (Table 1). Additionally the average read length after quality filtering (‘no primer’: 227 bp; ‘fuzzy match’: 220 bp; ‘perfect match’: 217 bp) was longer than the limit of the technology at the time of sequencing. The lengths of the usable sequences resulting from semiconductor sequencing therefore was comparable to other fungal environmental studies using 454 pyrosequencing [5], [17], [18], but at a similar or lower cost per usable sequence (IonTorrent: $0.01; 454: $0.01–$1.19; Table S4). As read lengths of the Ion Torrent at the time of writing has increased to 400 bp the sequence information compared to cost has increased further, likely making it even better suited to resolve fungal community composition.

The comparison of GC content with sequence quality in the sequences that were retained after quality filtering showed an apparent correspondence between regions of low GC content with low quality scores and this represents a potential weakness of the technology. Other Ion Torrent studies have shown a connection between GC content and sequence coverage [35], [36], although GC content alone appears not to be the solely responsible sequencing coverage issue [35]. The presence of GC-rich regions longer than 100 bp, mean GC content of the original sample and the organism sequenced influenced results either during PCR amplification in the library preparation or sequencing [35]. Although semiconductor sequencing has the potential to play a more prominent role in the study of fungal communities, the consequences of such shortcomings (common to many NGS technologies) on taxonomic assignment and on diversity estimates should be carefully considered.

Consensus concerning optimal sequence dissimilarity values corresponding to species-level differences has not yet emerged, though various grouping criteria have been applied (e.g., 97%: [3], [37]; 95%: [5], [38]; 90%: [16]). Such choices have important consequences for the ultimate description of taxon richness and community composition (Table 1). Considering the small amount of source material from which DNA was extracted relative to the vast number of OTUs recovered in our study, it is questionable whether all of them represent different species. Technical complications, including PCR artifacts and DNA polymerase fidelity, can lead to an artificial increase of OTUs [39], but these issues can be minimized using polymerases with proofreading capabilities. The large number of OTUs could also reflect diversity at lower levels than the species. Although fungal endophyte diversity is known to be high [15], [16], spatial structure of fungal communities within plants is largely unknown and no information on the numbers of individuals in a tree is available. In addition, OTU richness could actually reflect intra-genomic diversity of the ITS1 rDNA region. Intra-genomic sequence divergence among repetitive copies of the ITS has been documented previously [40], [41] and may be widespread [42]. The influence of low-abundance divergent copies of commonly-sequenced genes within the rDNA repeat region on estimates of OTU richness should likewise be carefully considered.

Advances in Ion Torrent and other NGS technologies will help to improve our understanding of microbial diversity across ecosystems and spatiotemporal scales. However, massive environmental sequencing projects often result in thousands of short DNA sequence reads which are deposited in public repositories including only limited taxonomic annotation. Our study highlights the value of curated databases, which form the cornerstone of metagenomic studies going forward and forge the invaluable link between sequence diversity and biological and ecological knowledge.

Despite the complications of taxonomic assignment, the value of the NGS approach is highlighted by the diversity of fungal families. Even given imperfect estimates of species-level diversity, highly diverse families from across the Kingdom Fungi co-occur within a few samples of plant tissue is truly an amazing finding. OTU richness was high within an exotically-grown *Eucalyptus* host (where as in non-native species, endophytic diversity might be predicted to be lower as has been observed with fungal and viral pathogens [43]). With respect to community composition, members of the Ascomycota and especially members of the Dothidiomycetes (including Mycosphaerellaceae, Botryosphaeriaceae, and Teratosphaeriaceae, groups which contain important known pathogens) were particularly dominant in our study (Figure 3). We also recovered a different community composition compared with that reported from traditional culture-dependent methods on *Eucalyptus* [44], [45]. Mycosphaerellaceae, which had many sequences in our study, was hardly present in these earlier studies and members of the Teratosphaeriaceae were not recovered at all (Table S5). Many families that were found in these earlier studies occurred also in our dataset, but their sequence abundance was often low. Valsaceae for example dominate the study by Fisher et al. (1996) but only few sequences of this family were recovered in our study and only with less stringent quality filtering. The only dominant family in our study that is commonly found in isolation studies of endophytes from healthy *Eucalyptus* tissue are the Botryosphaeriaceae [44]–[46], but with up to 31% of the read abundance they might be more prevalent in fungal communities than previously believed [47]–[49].

A few groups of fungi that are known to contain yeasts were also prevalent in this study, including the Sporidiobolales, Saccharomycetales, and Cystofilobasidiales. Many of these are regularly isolated from leaf surfaces [50], but they also have been recovered from within plant storage tissue such as potato and plum [51]. Whether some of these taxa recovered in our samples were surface inhabitants rather than true endophytes is difficult to say, as aggressive sterilization methods common in culturing studies is incompatible with sequencing as it is unknown whether this process degrades DNA.

In general, fungal diversity at the family level was similar across our filtering and clustering treatments, though the total number of sequences and OTUs differed markedly. However, important differences did exist. For example, the order Taphrinales was represented by a high richness of sequence reads in the ‘no primer’ approach, although the sequences were similar and this resulted in a low estimated number of unique OTUs (e.g., 95%: 6812 sequences/14 OTUs). *Post hoc* investigation of this pattern revealed that the ITS1 sequence for many groups exceeded the read lengths of the Ion Torrent PGM and were removed during filtering. We also found examples where the reverse primer sequence showed too many mismatches to be picked up by the quality filtering. Although read length issues in this case should be resolved with the 400 bp sequencing kit, applying a strict quality filtering approach has the potential to bias our interpretation of the importance of certain groups of fungi by removing them before the analyses.

Deep sequencing has revealed an enormous and previously undetected diversity of fungal endophytes and is currently the only feasible method to assess and compare such communities. Semiconductor sequencing such as the Ion Torrent technology utilized in this study displayed great potential for describing the vast diversity in plant associated fungal communities. To our knowledge this is the first study that demonstrates the feasibility of semiconductor sequencing in recovering fungal diversity. Different parameter settings in quality filtering resulted in differences in the richness of fungal families. Additionally, as others before us (e.g., [14]) we show that the vast and growing accumulation of sequences of unknown taxonomic affiliation present in public repositories blurs the actual known fungal family diversity associated with trees. These shortcomings, however, appear to be minor compared to the advantages that cost-effective NGS technologies now offer to compare whole communities over space and time.

## Supporting Information

Figure S1 Sequence read length histogram of the raw data.
(PDF)

Figure S2 Sequence read length histograms by quality filtering method.
(PDF)

Figure S3 Quality score reports for the quality filtering parameter settings. Top figure: average quality distribution. Bottom figure: distribution of sequence coverage for the mean average at the different bp positions.
(PDF)

Figure S4 Individual rarefaction curves for the three different quality parameter settings (‘no primer’, ‘fuzzy match’, ‘perfect match’) and the three OTU sequence similarity level values (90%, 95%, 97%) including the 95% confidence interval.
(PDF)

Figure S5 Mean log-transformed e-values of the BLAST comparison against the filtered and the unfiltered database by quality parameter setting and sequence similarity. Data shown for full dataset without removal of singletons.
(PDF)

Table S1 Sequence numbers and OTUs per family for the different quality filters and OTU similarity level values based on the BLAST comparison against the filtered database. Singleton OTUs and corresponding sequences were removed.
(XLSX)

Table S2 Family richness resulting from the different quality filters and OTU sequence similarity levels.
(XLSX)

Table S3 Taxa that appear only in the ‘no primer’ settings and 95% sequence similarity according to the respective pattern (Sequence too short, Mismatch in reverse primer sequence, Reverse primer sequence not found).
(XLSX)

Table S4 Comparison of costs, sequence output, cost per individual sequence and sequenced region between our study and selected fungal community studies using 454 pyrosequencing.
(XLSX)

Table S5 Comparison of our sequence based study of *Eucalyptus* endophytes to the earlier studies of Fisher et al. (1993) and Smith et al. (1996).
(XLSX)
