# Supplementary figures and images for: Ion Torrent PGM as Tool for Fungal Community Analysis: A Case Study of Endophytes in Eucalyptus grandis Reveals High Taxonomic Diversity
Source: PLoS One. 2013 Dec 16;8(12):e81718. doi: 10.1371/journal.pone.0081718 (PMC3864840; doi:10.1371/journal.pone.0081718)

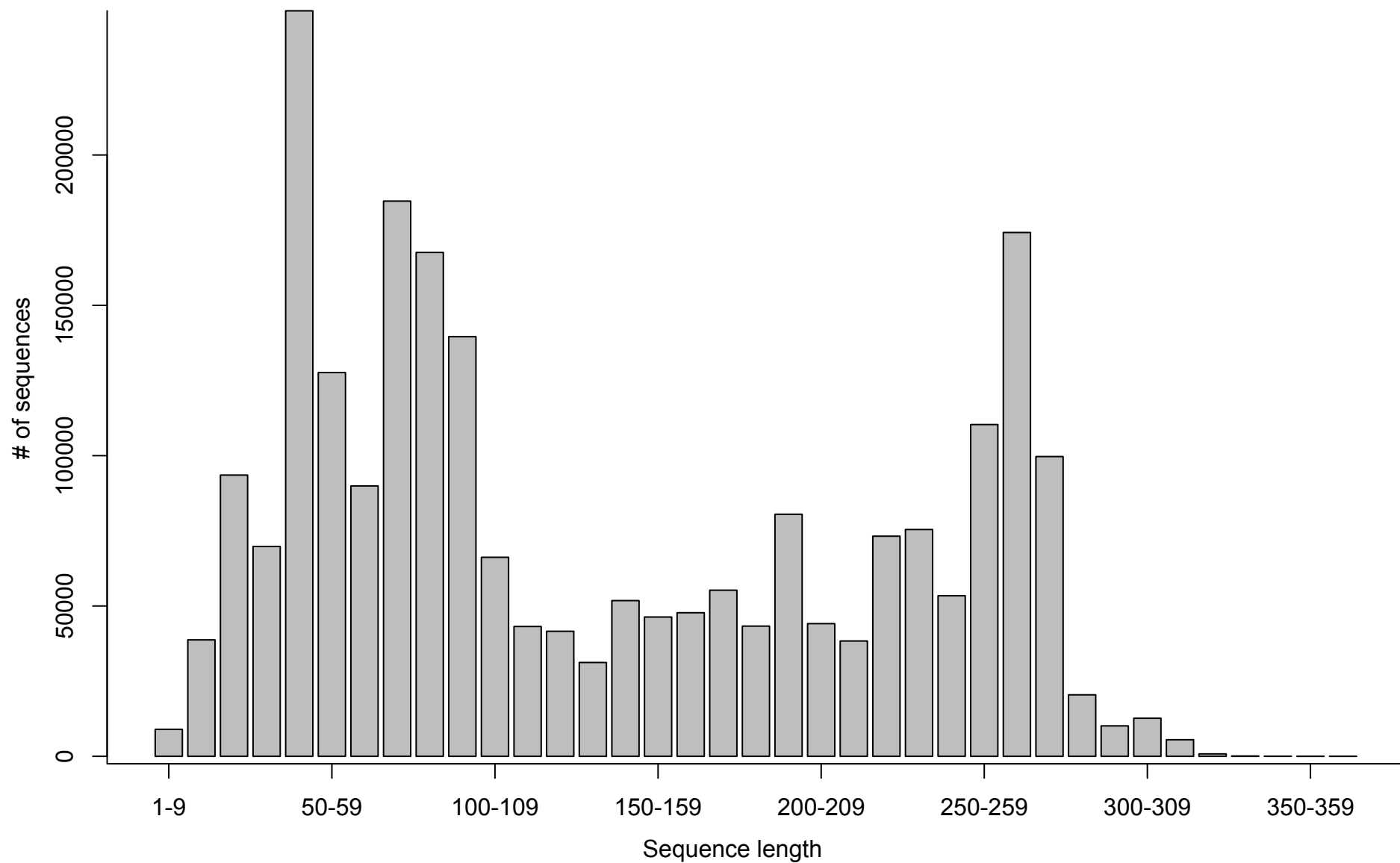

Supplement: Figure S1 — Sequence read length histogram of the raw data. (PDF) [file pone.0081718.s001.pdf]

a) 'no primer'

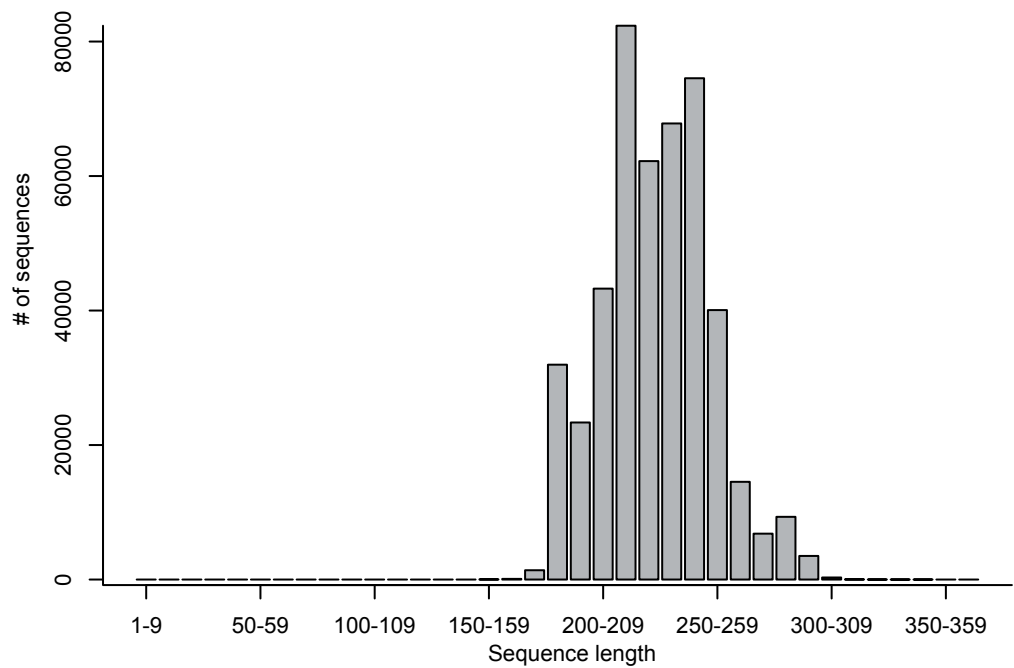

b) 'fuzzy match'

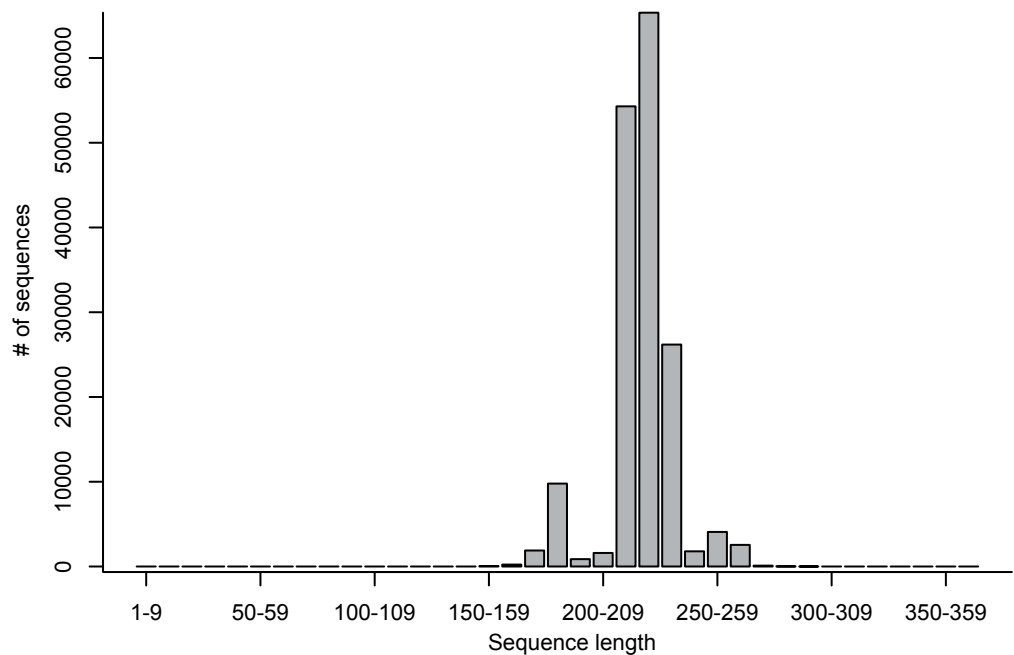

c) 'perfect match'

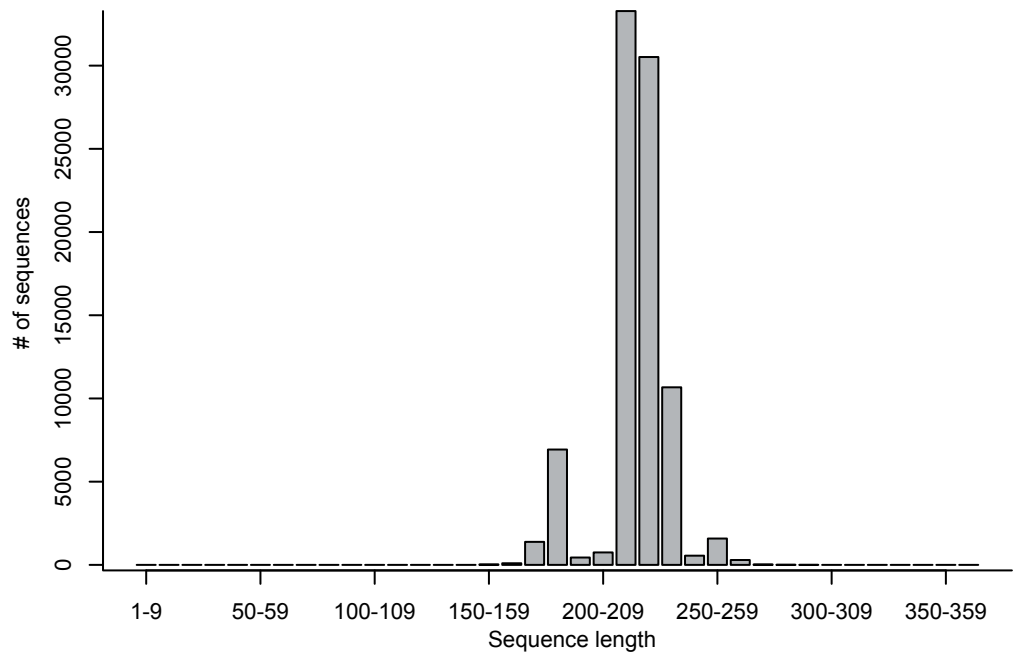

Supplement: Figure S2 — Sequence read length histograms by quality filtering method. (PDF) [file pone.0081718.s002.pdf]

‘no primer’

a) 90%

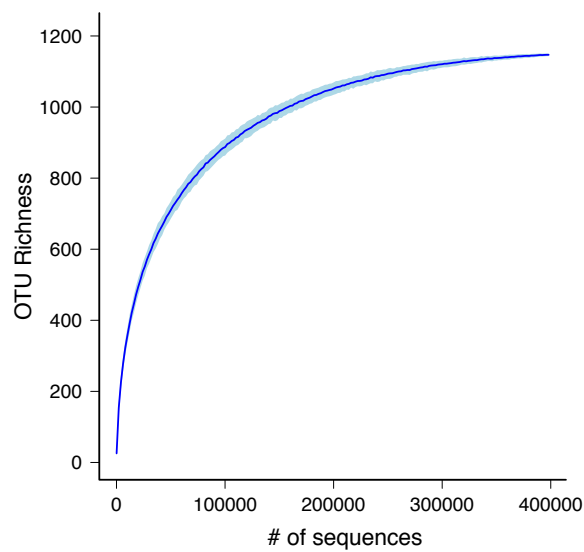

b) 95%

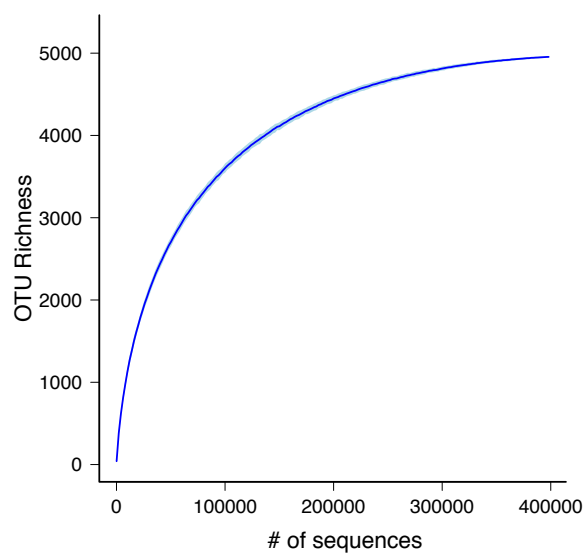

c) 97%

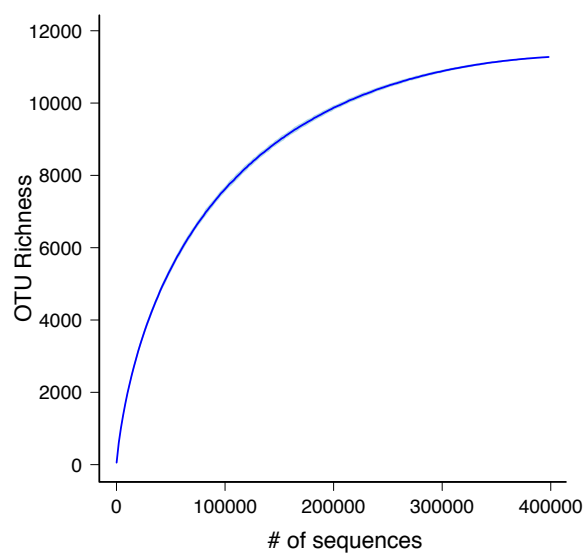

‘fuzzy match’

a) 90%

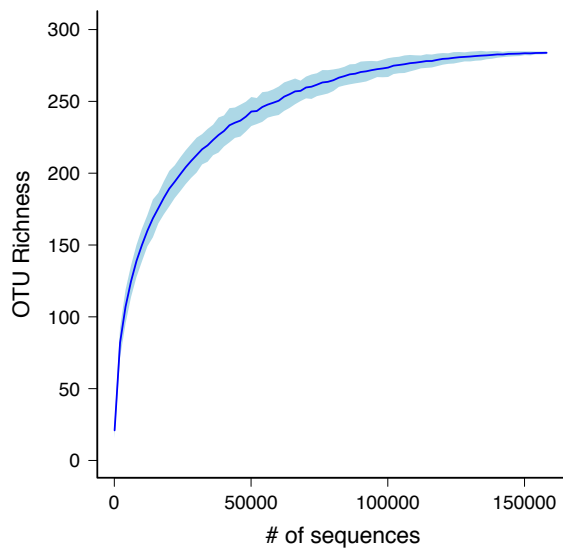

b) 95%

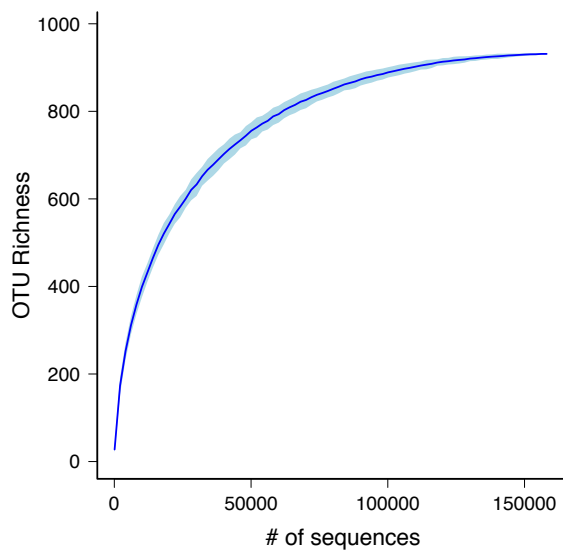

c) 97%

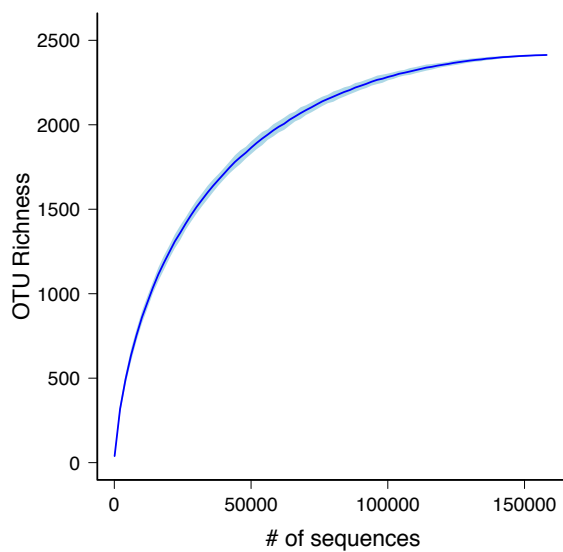

‘perfect match’

a) 90%

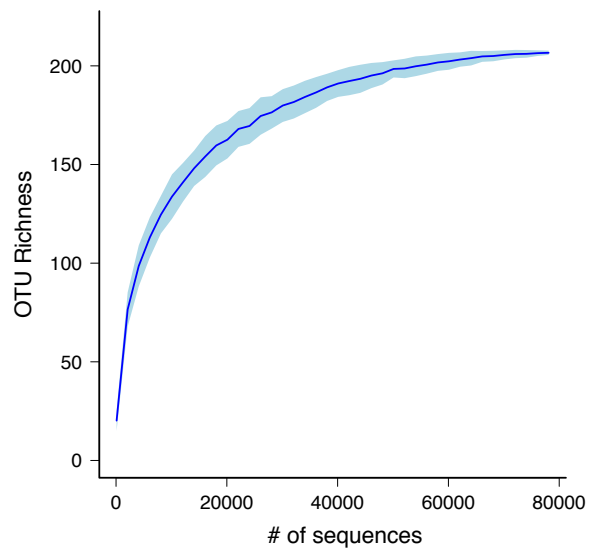

b) 95%

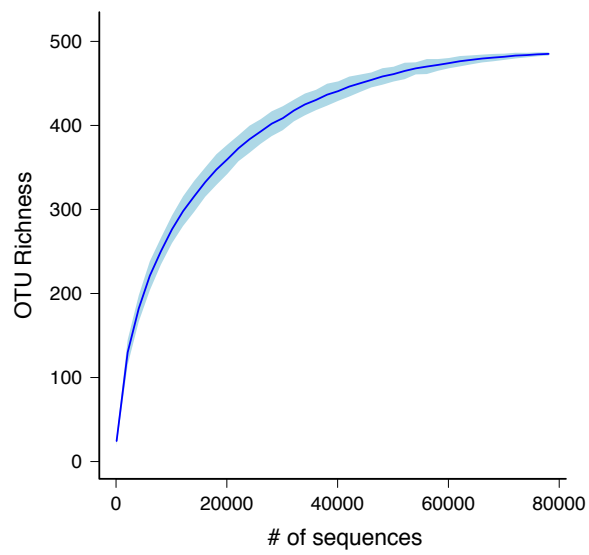

c) 97%

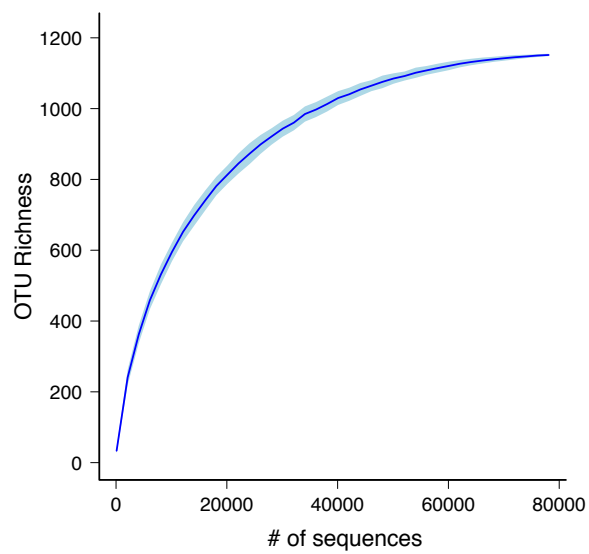

Supplement: Figure S4 — Individual rarefaction curves for the three different quality parameter settings (‘no primer’, ‘fuzzy match’, ‘perfect match’) and the three OTU sequence similarity level values (90%, 95%, 97%) including the 95% confidence interval. (PDF) [file pone.0081718.s004.pdf]
