# Supplementary material for: Ion Torrent PGM as Tool for Fungal Community Analysis: A Case Study of Endophytes in Eucalyptus grandis Reveals High Taxonomic Diversity
Source: PLoS One. 2013 Dec 16;8(12):e81718. doi: 10.1371/journal.pone.0081718 (PMC3864840; doi:10.1371/journal.pone.0081718)
Supplement: Figure S3 — Quality score reports for the quality filtering parameter settings. Top figure: average quality distribution. Bottom figure: distribution of sequence coverage for the mean average at the different bp positions. (PDF) [file pone.0081718.s003.pdf]

'perfect match'

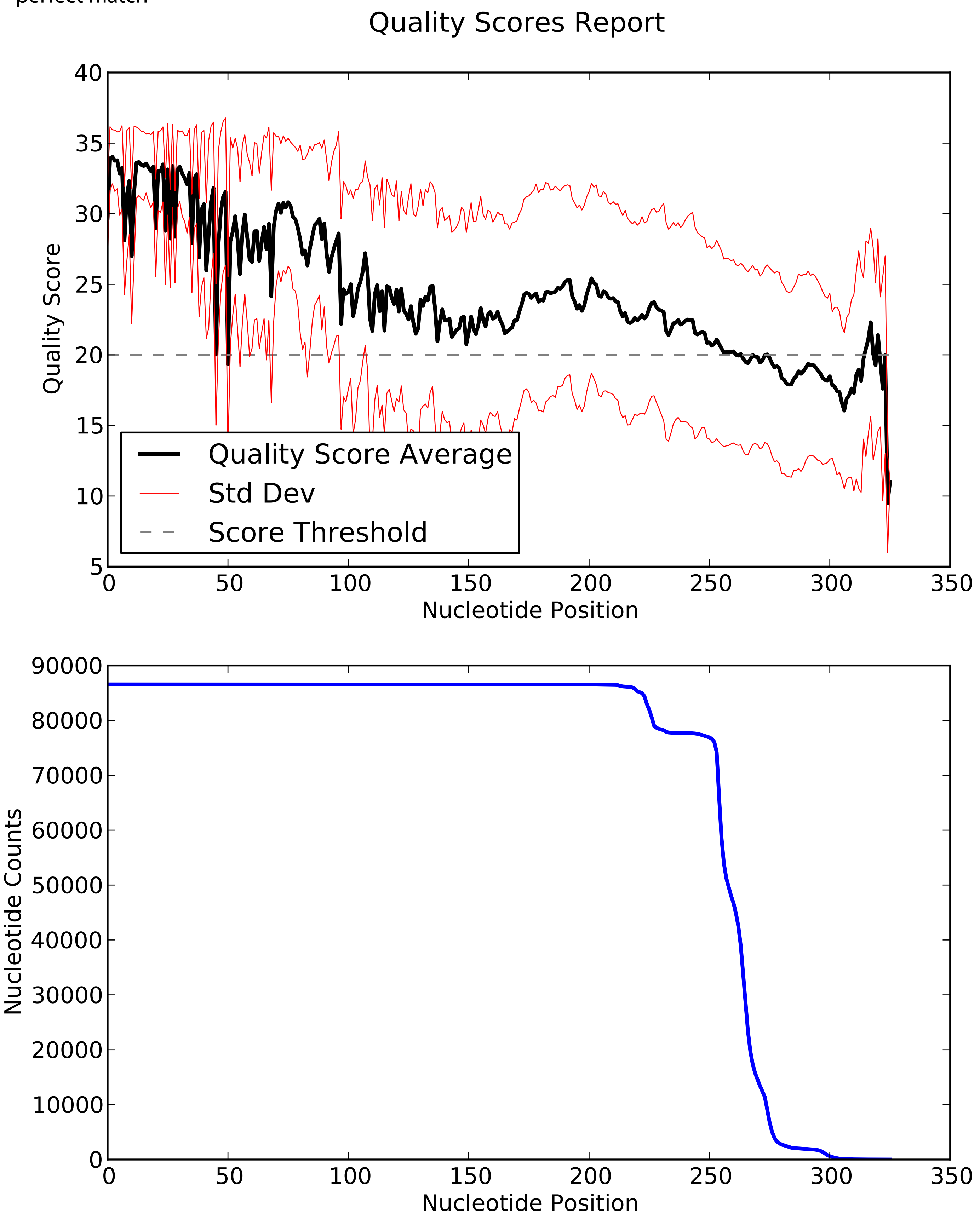

'fuzzy match'

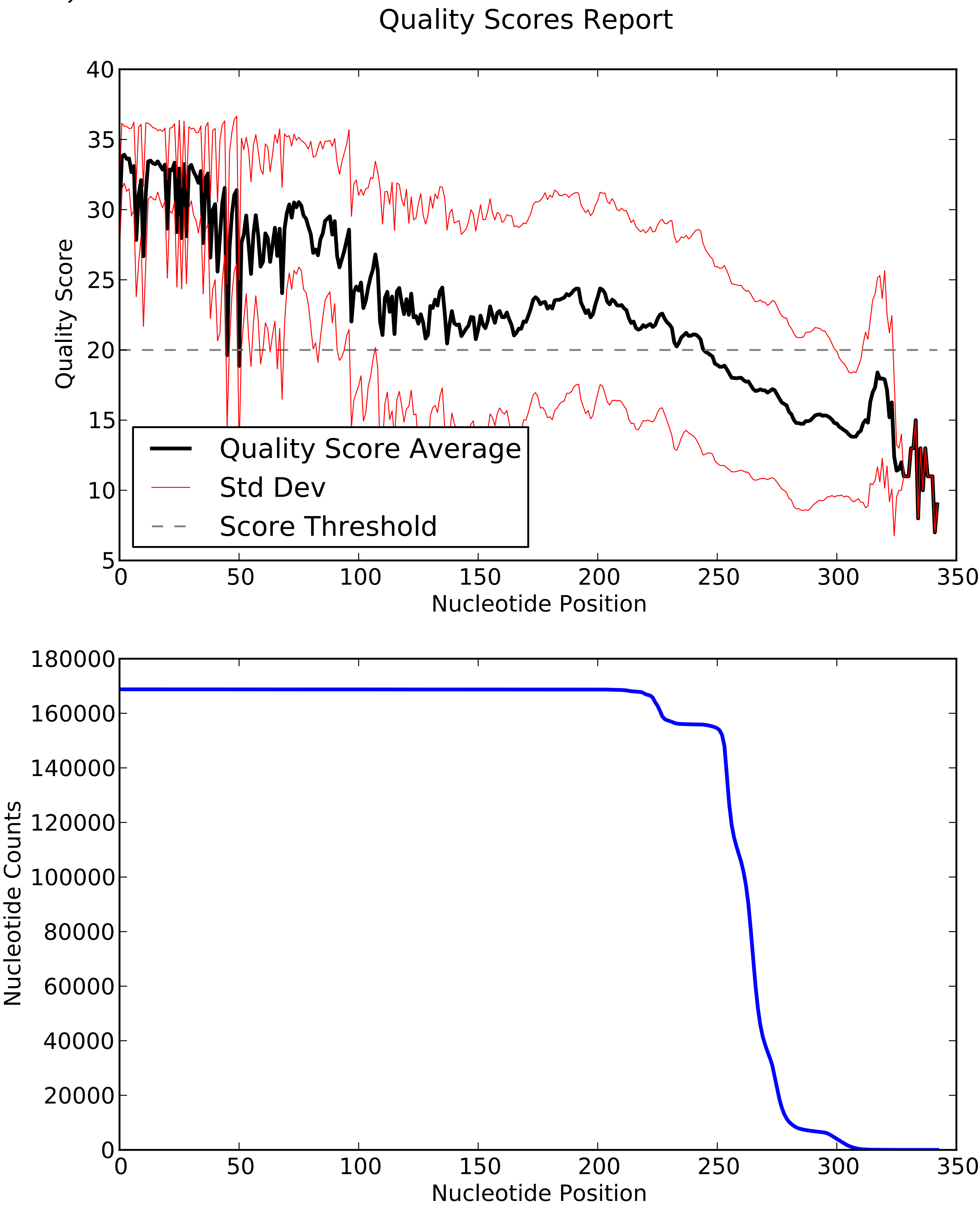
Quality Scores Report
Quality Score
Nucleotide Position
Quality Score Average
Std Dev
Score Threshold
0
5
10
15
20
25
30
35
40
50
100
150
200
250
300
350
Nucleotide Counts
Nucleotide Position
0
20000
40000
60000
80000
100000
120000
140000
160000
180000

'no primer'

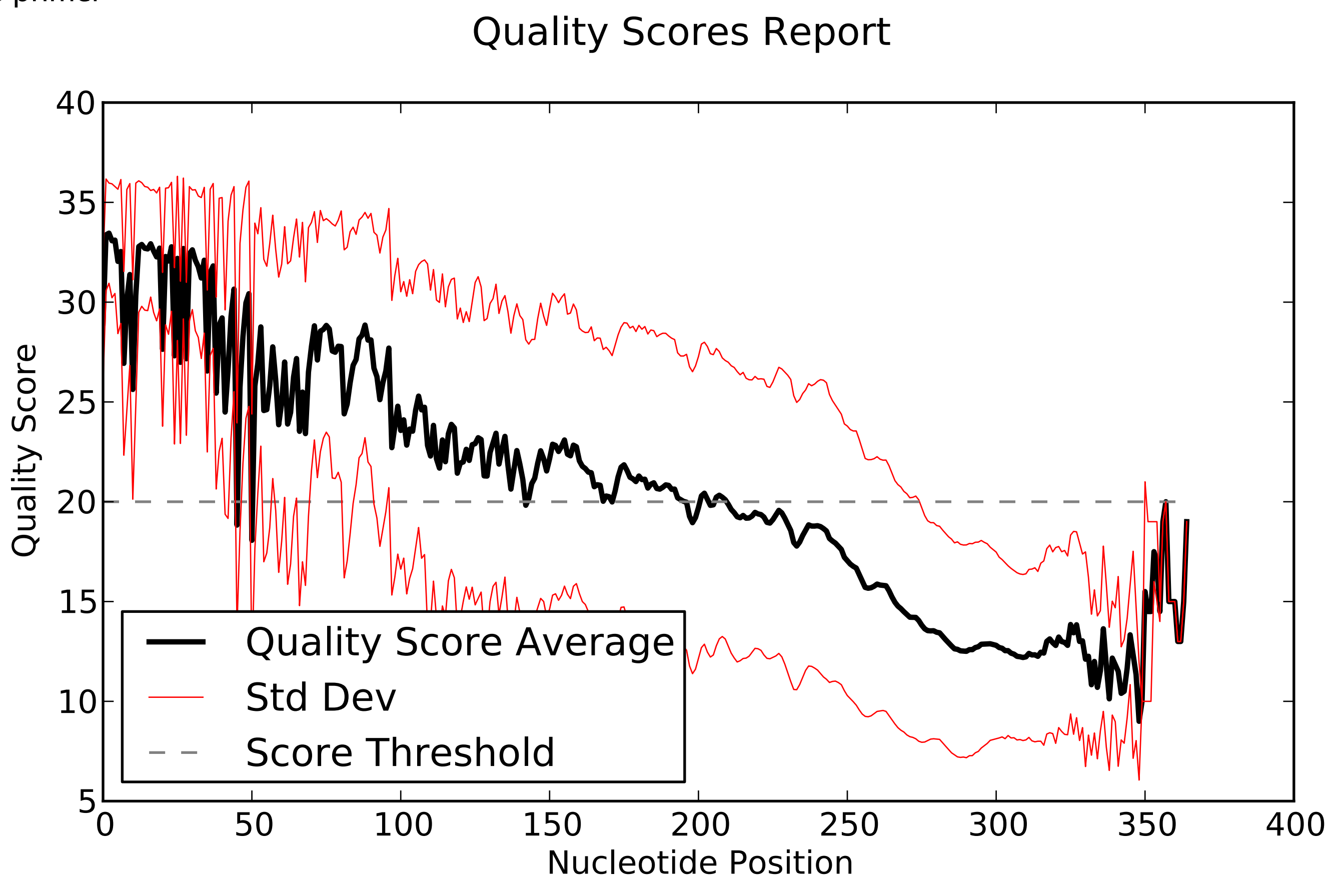
Quality Scores Report
Quality Score
Nucleotide Position
Quality Score Average
Std Dev
Score Threshold
40
35
30
25
20
15
10
5
0
50
100
150
200
250
300
350
400

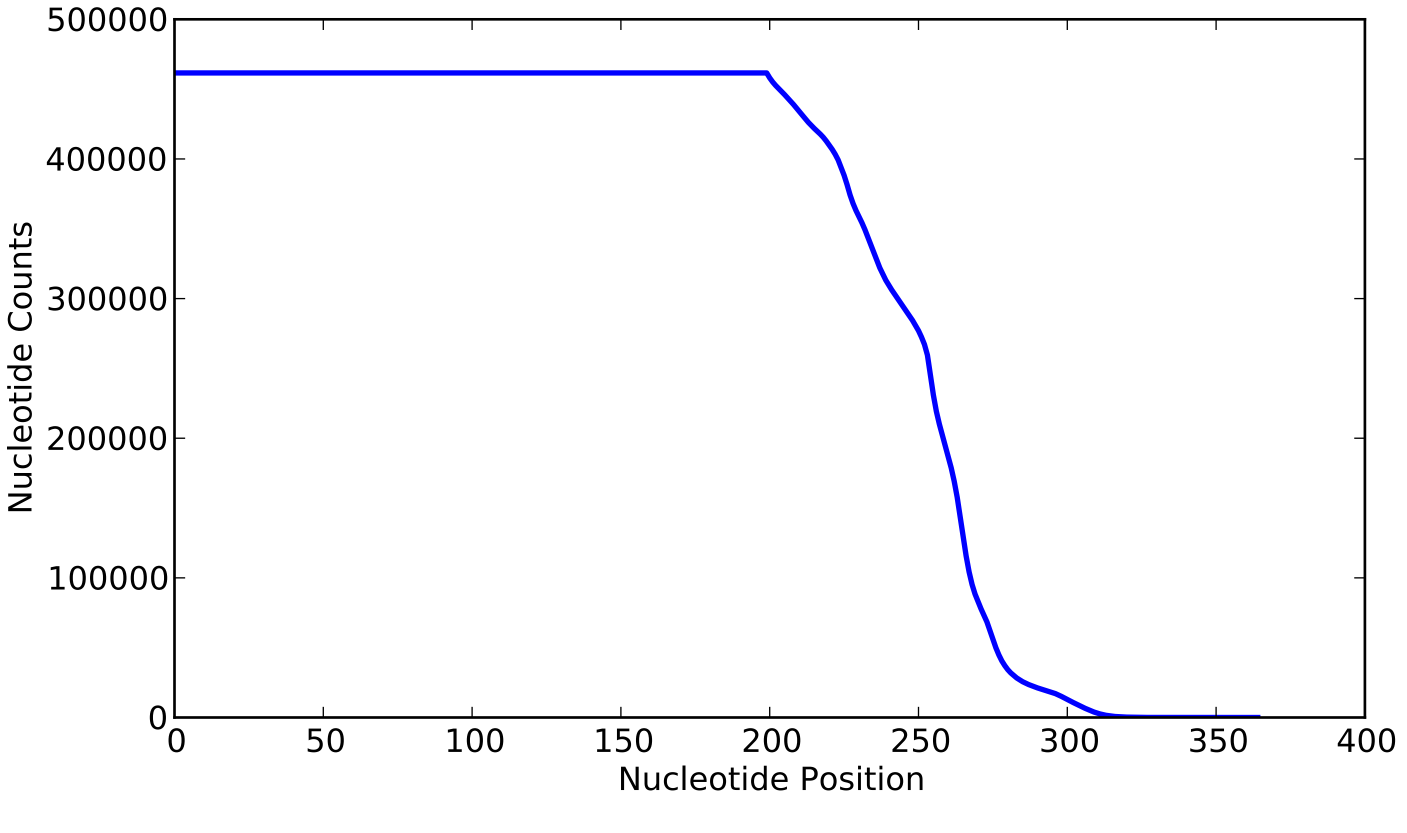
Nucleotide Counts
Nucleotide Position
500000
400000
300000
200000
100000
0
50
100
150
200
250
300
350
400
