# Supplementary material for: Ion Torrent PGM as Tool for Fungal Community Analysis: A Case Study of Endophytes in Eucalyptus grandis Reveals High Taxonomic Diversity
Source: PLoS One. 2013 Dec 16;8(12):e81718. doi: 10.1371/journal.pone.0081718 (PMC3864840; doi:10.1371/journal.pone.0081718)
Supplement: Figure S5 — Mean log-transformed e-values of the BLAST comparison against the filtered and the unfiltered database by quality parameter setting and sequence similarity. Data shown for full dataset without removal of singletons. (PDF) [file pone.0081718.s005.pdf]

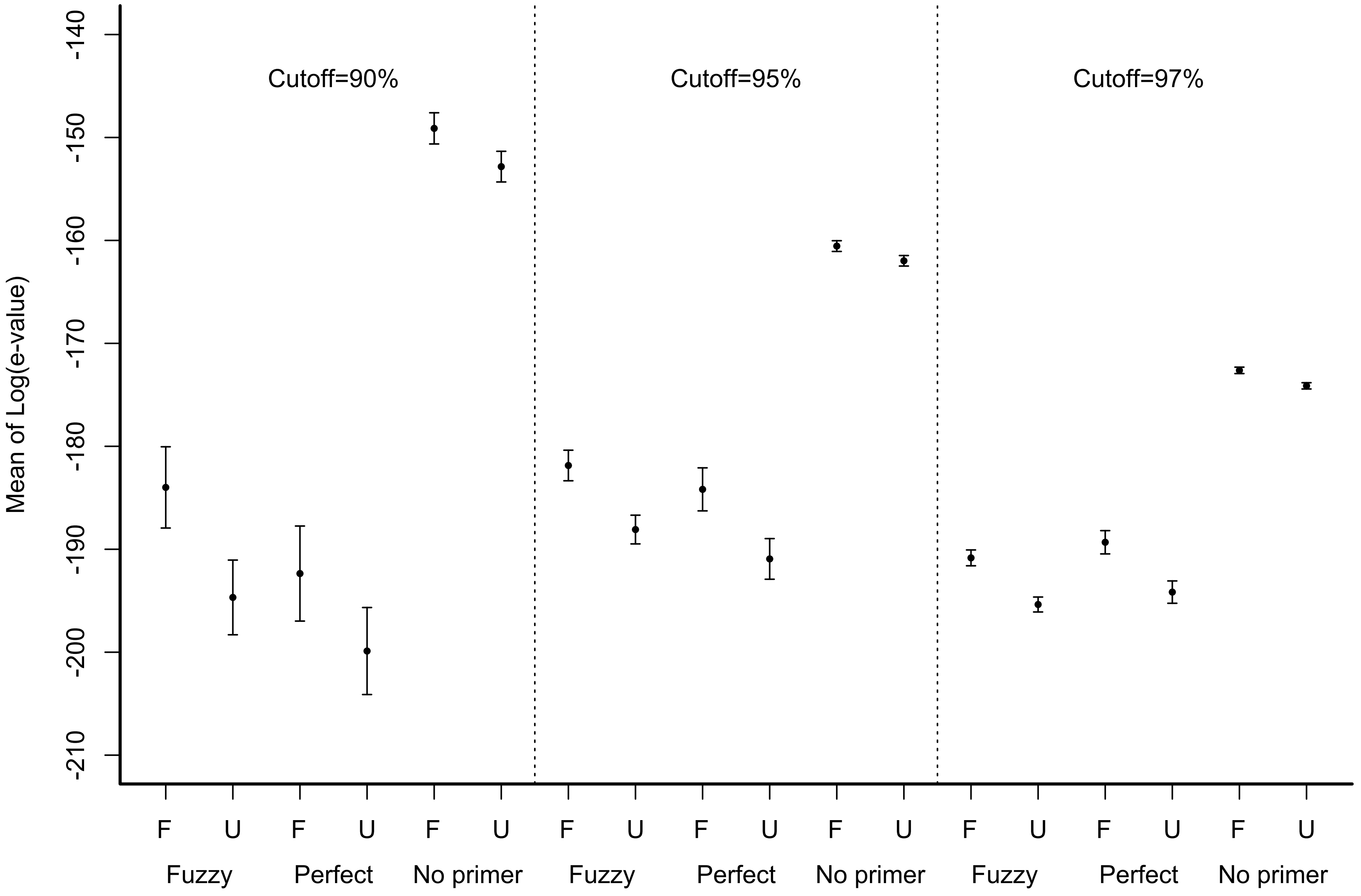

F = UNITE database filtered for sequences with insufficient taxonomy
U = unfiltered UNITE database
